# The prevalence of asymptomatic neurosyphilis among HIV-negative serofast patients in China: A meta-analysis

**DOI:** 10.1371/journal.pone.0241572

**Published:** 2020-11-04

**Authors:** Xutong Tan, Jiahui Zhang, Jing Li, Xiaoli Yue, Xiangdong Gong

**Affiliations:** 1 Department of STD Epidemiology, Institute of Dermatology, Chinese Academy of Medical Sciences and Peking Union Medical College, Nanjing, China; 2 National Center for STD Control, Nanjing, China; Jiangsu provincial Center for Disease Control and Prevention, CHINA

## Abstract

**Introduction:**

Neurosyphilis can occur at any stage of syphilis. After treatment, 30%-40% of syphilis patients remained serofast. But the prevalence of asymptomatic neurosyphilis (ANS) among serofast syphilis patients remains unclear. Untimely treatment or improper management for ANS may result in neurological complications. So we perform the meta-analysis to evaluate the prevalence of ANS cases among HIV-negative serofast syphilis patients for exploring their relationship and addressing their clinical management.

**Methods:**

We searched CNKI, Wan Fang, VIP, CBMdisc, PubMed, Embase and Medline from January 1^st^ 1990 to September 22^nd^ 2020 for both English and Chinese records. We strictly restrict the eligibility criteria. STROBE was used for reporting quality assessment. We examined forest plots and conducted both fix-effects and random-effects to estimate prevalence by R version 3.6.2/R studio 1.2.1335 statistical software packages META version 4.9–9. If appropriate, between-study heterogeneity was examined using the I^2^ statistic and subgroup analysis.

**Results:**

Of 77 screened records, 5 were included. The pooled prevalence of ANS among HIV-negative serofast syphilis patients was 13% (95% CI 3%-23%; I^2^ = 93% P<0.01, 417 people). The prevalence of ANS for the verified ANS classification definition was 3% (95% CI 0%-7%; I^2^ = 67% P = 0.08, two studies, 189 people), and 21% (95% CI 6%-36%; I^2^ = 86% P<0.01, three studies, 228 people) for the likely ANS classification. The prevalence of ANS among the serofast syphilis patients who were followed up for one year was 29% (95% CI 22%-36%; I^2^ = 0% P = 0.5, two studies, 167 people) and 5% (95% CI 0%-13%; I^2^ = 79% P = 0.03, two studies, 144 people) for two years. The prevalence in the studies from different geographical subgroups was as follows: 9% (95% CI 0%-19%; I^2^ = 82% P<0.01, three studies, 169 people) in South-central China, 6% (95% CI 1%-10%; one study, 106 people) in East China, and 30% (95% CI 23%-38%; one study, 142 people) in North China.

**Conclusion:**

This meta-analysis showed a high estimated prevalence of ANS in HIV-negative serofast syphilis patients, the prevalence of ANS among patients diagnosed with the verified ANS case definition is much lower than that for the likely ANS classification. It may be necessary to carry out nontreponemal test, protein test and leukocyte count for cerebrospinal fluid (CSF) in treated serofast patients for better clinical management to avoid neurological complications. The case classification definition of ANS is a key factor to evaluate the prevalence. Geographical heterogeneity needs more studies to detect. In future we need better-design studies to explore relationship between ANS and serofast status.

## Introduction

Syphilis is a systemic and chronic sexually transmitted disease, which caused by *Treponema pallidum* subspecies *pallidum*, can disseminate to any organ after infection, even damage the nervous and cardiovascular system [1]. The World Health Organization (WHO) estimated 6 million new cases of syphilis globally between 15 and 49 years old in 2016 [2]. Syphilis has been continually increasing in China and reached 36 cases per 100,000 population in 2018 [3], which has become a concerned public health problem. In 2010, the China’s Ministry of Health (MOH) officially launched the first national program specially and directly aimed at controlling syphilis: the National Plan for Prevention and Control of Syphilis in China (2010–2020) [4], which required to strengthen the screening and standard treatment of syphilis patients to stop transmission and reduce harm.

After the recommended syphilis therapy, 30% to 40% of syphilis patients remained serofast [5]. Serofast is a status where low-level of nontreponemal antibody titers persist without seroreversion following initial ≥ 4-fold decline after standard treatment [6, 7]. As a puzzling clinical problem for serofast, it is uncertain for both clinician and patient whether the persistent positive serological reaction indicates persistent foci of spirochetes or progressive syphilitic lesions or whether it reflects the persistence of regain in the circulating blood following therapy [5], and studies showed the effect of retreatment was limited [8, 9]. Some studies think that treponema infection of nervous system may be one of the important reasons for serofast status [10, 11], so it is necessary to understand the prevalence of neurosyphilis in serofast patients with treated syphilis.

Neurosyphilis can occur at any stage of syphilis. As the most common form of neurosyphilis, the asymptomatic neurosyphilis (ANS) is characterized by the abnormality of cerebrospinal fluid (CSF) with no symptoms or signs of involvement of the central nervous system [12, 13]. There were a few studies on the prevalence of ANS among serofast patients in the world, and most in HIV-positive serofast patients. In 2010, a study from Isreli showed 31% of HIV-positive serofast patients were neurosyphilis [11]. Another study from Poland in 2018 showed the prevalence of HIV-positive early syphilis serofast patients was 42% [14]. A study from China in 2017 showed the prevalence of ANS in HIV-negative serofast patients was 30% [15]. However, no systematic review was performed on the prevalence of ANS among serofast patients according to the Cochrane Library. HIV infection can also cause CSF abnormalities, which makes it hard to differentiate the effect of nervous invasion of syphilis among HIV co-infection syphilis patients. Therefore, it is necessary to obtain the prevalence of ANS in HIV-negative syphilis serofast group, which has not been well-established [16, 17]. There still exists controversy about undergoing lumbar puncture and CSF testing among HIV-negative serofast syphilis patients. Untimely treatment or improper management of ANS may result in neurological complications [18, 19]. Aqueous crystalline penicillin G is the recommended dosage for neurosyphilis patients, which is different from those without nervous infected patients [20].

Furthermore, two problems obstructed the estimation of the prevalence of neurosyphilis, one is the relatively small retrospective cohort studies whose case definitions differ, and the other is the lack of consistent reporting data [21]. In order to fill this gap and control the heterogeneity, we are going to collect existing studies with consistent reporting data and perform a meta-analysis to evaluate the prevalence of those studies on ANS among HIV-negative serofast syphilis patients in China, to guide the management of serofast syphilis patients for clinicians, and to understand their relationship between neurosyphilis and serofast status.

## Materials and methods

We developed a plan before data collection and data processing. Our study was not registered in the PROSPERO database. This study was reported according to Preferred Reporting Items for Systematic Reviews and Meta-Analysis (PRISMA) guidelines [22].

### Data sources and search strategy

Seven bibliographic databases, including China National Knowledge Infrastructure (CNKI), Wan Fang, VIP, China Biology Medicine disc (CBMdisc), PubMed, Embase and Medline, were used to search for publications regarding serofast patients with CSF outcomes in China from Janunary 1^st^ 1990 to September 22^nd^ 2020. We used the terms “Serofast” or “Seroresistance”, and “neurosyphilis” or “cerebrospinal fluid”, and “China” in English databases. “xueqingguding” or “xueqingdikang”, and “shenjingmeidu” or “naojiye” in Chinese databases.

### Eligibility criteria

Studies reporting the proportion of ANS among serofast patients, or the results of CSF testing of serofast patients, were included in this current study. All the patients were treated at least once before, no symptoms or signs, and underwent lumbar puncture to get CSF outcomes. And we restrict the number of tested serofast patients to at least 20. Considering the possible distribution differences between different countries, we limited our study in mainland China.

Studies which had HIV-positive patients were excluded. Those stated no definition or unclear criteria of ANS or serofast, were out of our study. Furthermore, review articles, case-control studies and book chapters were also excluded.

Serofast status was defined by persistently positive low-level nontreponemal antibody titers without seroreversion after initial ≥ 4-fold decline to avoid the situation of treatment failure or serological non-response [6, 7].

According to the neurosyphilis case definition from Centers for Disease Control and Prevention of America (CDC) [23], we defined the ANS cases into two categories or groups: verified and likely ANS cases. A verified ANS case was defined as a person with the reactive VDRL in CSF in the absence of grossly bloody contamination of the CSF without clinical symptoms of neurosyphilis; A likely case was defined as a person with an elevated CSF protein (>50mg/dL^2^) or leukocyte count (>5 WBC/mm^3^ CSF) in the absence of other known causes of these abnormalities without clinical symptoms of neurosyphilis.

### Study selection

Two researchers (XTT, JHZ) reviewed the records included independently, first of abstracts and titles and then of full text records. Differences were resolved by discussion or adjudication by a third reviewer (XDG). We searched for all relevant studies and supplemented the search by screening bibliographies of identified articles. If two or more studies were published based on the same sample, the article with the greatest epidemiological quality was included. Moreover, we did not contact the authors of original studies for additional information. The full citation screening process is detailed using the PRISMA flow diagram.

### Quality assessment

The quality of included studies and risk of bias were assessed using the STROBE checklist for observational studies reporting [24].

### Data extraction

For each record we extracted first author, publication year, study period, CSF testing methods for ANS, total number of serofast syphilis patients and ANS cases. We also extracted some factors such as sex, age, geographical region, serum RPR titers and follow-up time among original samples.

### Statistical methods

The pooled prevalence of ANS among HIV-negative serofast syphilis patients was calculated upon the number of receiving CSF testing patients with a meta-analysis. Fix-effects and random-effects models by the τ^2^ estimator were both used to calculate and prepare forest plots using R version 3.6.2/R studio 1.2.1335 statistical software packages META version 4.9–9. Higgins inconsistency test (I^2^) was used to assess heterogeneity with the percentage of observed variation across studies. The potential sources of heterogeneity were further investigated using subgroup analysis. The factors investigated comprised the geographical region, case classification and follow-up time. To examine the potential publication bias, we used the funnel plot and tested using Egger’s test. A P<0.05 was considered to be statistically significant.

## Results

### Included studies

In this study, a total of 148 papers were returned by the search, and 107 articles were excluded by duplicates and abstracts, 36 studies were excluded based on full-text review. Only 5 studies were included for the final analysis which met the eligibility criteria. The flowchart of reviews showed the detailed process of selection (S1 Fig).

### Quality assessment

All of the included articles were assessed for quality with the STORBE in S2 Checklist. All of the studies gave adequate source of population and study time. All studies had follow-up at least for one year. The inclusion and exclusion had made clear despite most of them were referring to previous publications. There were 3 studies presenting specific testing outcomes for us to assurance the quality [15, 25, 26]. None stated details about handling the missing data and confounding.

### Study characteristics

Table 1 summarised the characteristics of studies included in this review [15, 25–28]. Five studies published from 2009 to 2017, comprised of 417 serofast patients. All studies were retrospective cross-sectional and hospital-based. There were two studies published in English [15, 25], and three in Chinese [26–28]. Two studies showed the same median age in 30-year old [26, 27]. One study demonstrated as high as 72% of female cases and the serum RPR titers of all serofast patients after treatment were ≤1:4 [26] (Table 1).

**Table 1:** Characteristics of studies included in the meta-analyses.

Study	Language	Province, Region	Case Classification	No. of ANS	No. of serofast patients	Prevalence of ANS	Stage of pre-treated syphilis	Median serofast patient age (years)	follow-up time
*Zhu et al. (2009) [26]	Chinese	Hunan, South-central China	Likely: elevated CSF protein or (and) WBC	6	25	24.00%	36% with early syphilis, 64% with late syphilis	30.9	1 year
Lin et al. (2010) [27]	Chinese	Guangdong, South-central China	Verified: reactive VDRL in CSF	1	83	1.20%	100% with latent syphilis	34.2	2 years
Zhou et al. (2012) [25]	English	Shanghai, East China	Verified: reactive VDRL in CSF	6	106	5.70%	100% with secondary syphilis	NM	NM
Zheng et al. (2016) [28]	Chinese	Guangdong, South-central China	Likely: elevated CSF protein or (and) WBC	6	61	9.80%	NM	NM	2 years
Cai et al. (2017) [15]	English	Beijing, North China	Likely: elevated CSF protein or (and) WBC	43	142	30.30%	NM	NM	1 year

Prevalece of ANS, the prevalence of asymptomatic neurosyphilis among serofast syphilis patients; NM, not mentioned;

*This study stated the serum RPR titers of all serofast patients were ≤1:4 with a male to female ratio at 7/18.

Diagnostic classification: the likely ANS case is using elevated CSF protein (>50mg/dL^2^) or leukocyte count (>5 WBC/mm^3^ CSF) in the absence of other known cause of these abnormalities, the verified ANS case is using a reactive VDRL in CSF.

### Overall pooled prevalence of ANS among HIV-negative serofast syphilis patients

5 studies with 417 serofast patients were included. The prevalence of five studies was from 1% to 30%. The pooled prevalence of ANS among HIV-negative serofast patients was 13.0% (95%CI 3%-23%; I^2^ = 93%; P<0.01) with high significant level of heterogeneity for combined effect size by using random effects model (S2 Fig).

### Subgroup analysis

#### Case classification definition level

Due to the heterogeneity of case classification, random effects model was performed in the subgroup analysis. There were two case classification categories among the included studies, verified ANS cases were defined by a reactive VDRL in CSF, and likely ANS cases were defined by elevated WBC or protein in CSF. The pooled prevalence of studies using the verified ANS classification was 3% (95%CI 0%-7%; I^2^ = 67% P = 0.08). The pooled prevalence of studies using the likely ANS classification was 21% (95%CI 6%-36%; I^2^ = 86% P<0.01) (S3 Fig).

#### Follow-up time

Follow-up time can be divided to one to two years. The prevalence for one year was 29% (95%CI 22%-36%; I^2^ = 0% P = 0.5, two studies) by fixed effects model with no heterogeneity, and the prevalence for two years was 5% (95%CI 0%-13%; I^2^ = 79% P = 0.03, two studies) by random effects model (S4 Fig).

#### Different regions

Random effects model was preferred in this subgroup analysis. The prevalence in South-central China was 9% (95%CI 0%-19%; I^2^ = 82% P<0.01), one was 24.0% conducted in Hunan, other two both conducted in Guangdong were 1.2% and 9.8% respectively. One study performed in Shanghai, East China, was 5.7%. And one in Beijing, North China, was 30.3% (S5 Fig).

### Publication bias

Funnel plot graphic and egger’s regression asymmetry test showed no statistically significance over five studies (t = 2.37, P = 0.098) (S6 Fig).

## Discussion

Only five studies were included in this meta-analysis which met the selection criteria, and the results showed the pooled prevalence of ANS among HIV-negative serofast syphilis patients was high. A number of studies were ruled out at the screening records phrase, because some didn’t have clear case definition, some didn’t consider the effect of past treatment. So we strictly restricted our eligibility criteria and excluded both treatment failure and serological non-response patients from the serofast status.

Subgroup analysis showed follow-up time and case classification definition had great impact on the pooled prevalence. Future research should be focused on developing or employing a standardized definition of syphilis serofast and case classification criteria of ANS. When follow-up time was prolonged, the prevalence would drop greatly. It might be associated with the case classification definition, because the studies following up for one year were both using the likely ANS case definition. The prevalence of those using the likely ANS case definition was higher than that for the verified ANS case definition. Case classification definition is related to the specificity and sensitivity of diagnostic techniques. The specificity of the verified ANS case classification using VDRL testing is proved to be very high (91.6%-100%), the sensitivity of the CSF-VDRL is not enough high with range varying from 1.5% to 69% [29–32], which might underestimate the prevalence of ANS. As lumbar puncture is already an invasive procedure, we might suggest a higher sensitive case definition to avoid a missed diagnosis, especially among ANS. Currently there is no single highly sensitive and specific diagnostic test existed for neurosyphilis, the diagnosis depends on the clinical findings and CSF abnormalities as well as clinical judgment, but also this needs a deep understanding of the disease spectrum and the strengths and limitations of diagnostic techniques [33]. The CSF abnormality is not only a sign of neuroinvasion, but also for making a judgment on the success or failure of treatment. CSF testing results have a guiding role for clinicians in the selection of further treatment. For those neurosyphilis patients, aqueous crystalline penicillin G would be supposed to be used rather than benzathine penicillin G [20].

Subgroup analysis also found the heterogeneity existed between different regions. Since only one study was included in both North and East China, our study cannot draw the conclusion whether the prevalence in the north region is highest or not. It is necessary to carry out more relevant studies in this region to confirm the rate. In addition, our included studies were from Beijing, Shanghai, Guangdong, and Hunan province, no related studies from other provinces or cities, the pooled prevalence did not reflect the situation of the whole China.

Egger’s test didn’t indicate any evidence of publication bias of all. It is necessary of regular follow-up to avoid the possibility of the process developing into symptomatic neurosyphilis among those serofast patients.

There are some limitations to this meta-analysis. First, only five studies met inclusion and exclusion criteria. Second, limited characteristics were described in those included studies, thus we did not get more reliable information of relevant risk factors between serofast syphilis patients and neurosyphilis. Future it is still necessary to prolong follow-up time among serofast patients because it is better to resolve early neurosyphilis clinical abnormalities than late. It is supposed to have more well-designed studies to better describe the prevalence and risk factors between ANS and serofast status, such as the follow-up time, the success or failure outcome of treatment, their basic chronic disease situation and so on.

## Conclusions

The pooled prevalence of ANS among HIV-negative serofast patients was high. The case classification definition of ANS is a key factor to evaluate the prevalence, the prevalence of those diagnosed with the verified ANS classification definition is much lower than that for the likely ANS classification. It may be necessary to carry out nontreponemal test, protein test and leukocyte count for cerebrospinal fluid (CSF) in treated serofast patients for better clinical management to avoid neurological complications. Geographical heterogeneity needs more studies to detect. In future study, consistent definition of serofast status should be unified, and more well-designed studies are needed for detecting relationship between neurosyphilis and serofast status.

## Supporting information

S1 Checklist PRISMA checklist. (DOC)

S2 Checklist STROBE checklist. (DOC)

S1 Protocol Meta-analysis protocol (Chinese version). (DOCX)

S1 Appendix Search strategy. (DOCX)

S1 Fig PRISMA flow chart. (TIF)

S2 Fig Forest plot showing the pooled prevalence of asymptomatic neurosyphilis. (TIF)

S3 Fig Prevalence estimates stratified by case classification. (TIF)

S4 Fig Prevalence estimates stratified by follow-up time. (TIF)

S5 Fig Prevalence estimates stratified by regions. (TIF)

S6 Fig Funnel plot. (TIF)

## References

[1] Singh AE, Romanowski B. Syphilis: review with emphasis on clinical, epidemiologic, and some biologic features. Clin Microbiol Rev. 1999 4; 12(2):187–209. . 10194456 10.1128/cmr.12.2.187 PMC88914

[2] World Health Organization. (2018). Report on global sexually transmitted infection surveillance 2018. World Health Organization. https://apps.who.int/iris/handle/10665/277258.

[3] Chinese Center for Disease Control and Prevention. An overview of the epidemic situation of notifiable infectious diseases in China in 2018. 2019; http://www.nhc.gov.cn/jkj/s3578/201904/050427ff32704a5db64f4ae1f6d57c6c.shtml.

[4] Notice of the Ministry of Health on issuing national program for prevention and control of syphilis in China (2010–2020): China Ministry of Health 2010. 2010; http://www.gov.cn/gzdt/2010-06/21/content_1632301.htm.

[5] Qin J, Yang T, Wang H, Feng T, Liu X. Potential predictors for serofast state after treatment among HIV-negative persons with syphilis in China: A systematic review and meta-analysis. Iran J Public Health. 2015 2;44(2):155–69. . 25905049 PMC4401873

[6] Clement ME, Okeke NL, Hicks CB. Treatment of syphilis: a systematic review. JAMA. 2014 11 12;312(18):1905–17. 10.1001/jama.2014.13259 . 25387188 PMC6690208

[7] Seña AC, Zhang XH, Li T, Zheng HP, Yang B, Yang LG et al A systematic review of syphilis serological treatment outcomes in HIV-infected and HIV-uninfected persons: rethinking the significance of serological non-responsiveness and the serofast state after therapy. BMC Infect Dis. 2015 10 28;15:479 10.1186/s12879-015-1209-0 . 26511465 PMC4625448

[8] Seña AC, Wolff M, Behets F, Van Damme K, Martin DH, Leone P et al Response to therapy following retreatment of serofast early syphilis patients with benzathine penicillin. Clin Infect Dis. 2013 2;56(3):420–2. 10.1093/cid/cis918 Epub 2012 Nov 1. . 23118269 PMC3590030

[9] Wang ZS, Liu XK, Li J. Serological response to therapy following retreatment of serofast early syphilis patients with benzathine penicillin. J Antimicrob Chemother. 2018 5 1;73(5):1348–1351. 10.1093/jac/dky006 . 29394361

[10] Serragui S, Yahyaoui M, Hassar M, Chkili T, Bouhaddioui N, Soulaymani R. A comparison study of two therapeutic protocols for neurosyphilis. Therapie. 1999 Sep-Oct;54(5):613–21. . 10667099

[11] Agmon-Levin N, Elbirt D, Asher I, Gradestein S, Werner B, Sthoeger Z. Syphilis and HIV co-infection in an Israeli HIV clinic: incidence and outcome. Int J STD AIDS. 2010 4;21(4):249–52. 10.1258/ijsa.2009.009011 . 20378895

[12] O’Leary PA CH, Moore JE, et al Cooperative clinical studies in the treatment of syphilis: Asymptomatic neurosyphilis. Arch Derm Syphilol. 1937;35:387–401.

[13] Lukehart SA, Hook EW 3rd, Baker-Zander SA, Collier AC, Critchlow CW, Handsfield HH. Invasion of the central nervous system by Treponema pallidum: implications for diagnosis and treatment. Ann Intern Med. 1988 12 1;109(11):855–62. 10.7326/0003-4819-109-11-855 . 3056164

[14] Pastuszczak M, Sitko M, Bociaga-Jasik M, Kucharz J, Wojas-Pelc A. Lack of antiretroviral therapy is associated with higher risk of neurosyphilis among HIV-infected patients who remain serofast after therapy for early syphilis. Medicine (Baltimore). 2018 11;97(45):e13171 10.1097/MD.0000000000013171 . 30407349 PMC6250445

[15] Cai SN, Long J, Chen C, Wan G, Lun WH. Incidence of asymptomatic neurosyphilis in serofast Chinese syphilis patients. Sci Rep. 2017 11 13;7(1):15456 10.1038/s41598-017-15641-w . 29133821 PMC5684362

[16] JE M. Asymptomatic neurosyphilis: A comparison of early and late asymptomatic neurosyphilis. Arch Derm Syphilol. 1928;18:99–108.

[17] Tong ML, Lin LR, Liu GL, Zhang HL, Zeng YL, Zheng WH et al Factors associated with serological cure and the serofast state of HIV-negative patients with primary, secondary, latent, and tertiary syphilis. PLoS One. 2013 7 23;8(7):e70102 10.1371/journal.pone.0070102 . 23894598 PMC3720935

[18] Moskovitz BL, Klimek JJ, Goldman RL, Fiumara NJ, Quintiliani R. Meningovascular syphilis after ‘appropriate’ treatment of primary syphilis. Arch Intern Med. 1982 1;142(1):139–40. . 7053714

[19] Wilner E, Brody JA. Prognosis of general paresis after treatment. Lancet. 1968 12 28;2(7583):1370–1. 10.1016/s0140-6736(68)92674-3 . 4177933

[20] Workowski KA, Bolan GA; Centers for Disease Control and Prevention Sexually transmitted diseases treatment guidelines, 2015. MMWR Recomm Rep 2015; 64:40–1. PMC5885289 26042815

[21] Tuddenham S, Ghanem KG. Neurosyphilis: Knowledge gaps and controversies. Sex Transm Dis. 2018 3; 45(3):147–151. 10.1097/OLQ.0000000000000723 . 29420441 PMC5808573

[22] Moher D, Liberati A, Tetzlaff J, Altman DG (2009). Preferred reporting items for systematic reviews and meta-analyses: the PRISMA statement. PLOS Med 6:e1000097 10.1371/journal.pmed.1000097 19621072 PMC2707599

[23] Centers for Disease Control and Prevention: Atlanta, GA: Department of Health and Human Services. Sexually transmitted disease surveillance 2018. 2019.

[24] von Elm E AD, Egger M, Pocock SJ, Gøtzsche PC, Vandenbroucke JP. The strengthening the reporting of observational studies in epidemiology (STROBE) statement: guidelines for reporting observational studies. PLoS Med. 2007;4(10):e296 10.1371/journal.pmed.0040296 17941714 PMC2020495

[25] Zhou P, Gu X, Lu H, Guan Z, Qian Y. Re-evaluation of serological criteria for early syphilis treatment efficacy: progression to neurosyphilis despite therapy. Sex Transm Infect. 2012 8;88(5):342–5. 10.1136/sextrans-2011-050247 Epub 2012 Feb 23. . 22363023

[26] Zhu JM. Test of cerebrospinal fluid in serofast syphilis patients. M.Sc. Thesis, Central South University. 2009; https://kns.cnki.net/KCMS/detail/detail.aspx?dbname=CMFD2010&filename=2009239801.nh.

[27] Lin LY, Yang RD, Zhang XB, Song WZ, He YQ, Liang YH et al Analysis on the prevalence of neurosyphilis in latent syphilis patients. Int J Dermatol Venereol. 2010;36 Epub 20110425. 10.3760/cma.j.issn.1673-4173.2010.06.002

[28] Zheng TH, Zeng TS, Feng TJ, Wu XB, Qiu LX. Cost-effectiveness analysis of neurosyphilis screening in serofast patients. Chinese Journal of Health Statistics. 2016;33.

[29] Davis AP, Stern J, Tantalo L, Sahi S, Holte S, Dunaway S, et al How well do neurologic symptoms identify individuals with neurosyphilis. Clin Infect Dis. 2018 1 18;66(3):363–367. 10.1093/cid/cix799 . 29020214 PMC5848223

[30] Nayak S, Acharjya B. VDRL test and its interpretation. Indian J Dermatol. 2012 1;57(1):3–8. 10.4103/0019-5154.92666 . 22470199 PMC3312652

[31] Ho EL, Tantalo LC, Jones T, Sahi SK, Marra CM. Point-of-care treponemal tests for neurosyphilis diagnosis. Sex Transm Dis. 2015 1;42(1):48–52. 10.1097/OLQ.0000000000000222 . 25504301 PMC4268876

[32] Davis LE, Schmitt JW. Clinical significance of cerebrospinal fluid tests for neurosyphilis. Ann Neurol. 1989 1;25(1):50–5. 10.1002/ana.410250108 . 2643918

[33] Marra CM. Neurosyphilis. Continuum (Minneap Minn). 2015 12;21(6 Neuroinfectious Disease):1714–28. 10.1212/CON.0000000000000250 . 26633785

