# Supplementary material for: The prevalence of asymptomatic neurosyphilis among HIV-negative serofast patients in China: A meta-analysis
Source: PLoS One. 2020 Nov 4;15(11):e0241572. doi: 10.1371/journal.pone.0241572 (PMC7641405; doi:10.1371/journal.pone.0241572)
Supplement: S2 Checklist — (DOC) [file pone.0241572.s002.doc]

**S2 Checklist. STROBE Assessment-Checklist of items that should be included in reports of *cross-sectional studies***

|  | Item No | Recommendation | **Zhu,**  **2009** | **Lin,**  **2010** | **Zhou,**  **2012** | **Zheng,**  **2016** | **Cai,**  **2017** |
| --- | --- | --- | --- | --- | --- | --- | --- |
| **Title and abstract** | 1 | (*a*) Indicate the study’s design with a commonly used term in the title or the abstract | N | Y | N | Y | N |
|  | 2 | (*b*) Provide in the abstract an informative and balanced summary of what was done and what was found | Y | Y | Y | Y | Y |
| Introduction | | | | | | | |
| Background/rationale | 3 | Explain the scientific background and rationale for the investigation being reported | Y | Y | Y | Y | Y |
| Objectives | 4 | State specific objectives, including any prespecified hypotheses | Y | Y | Y | Y | Y |
| Methods | | | | | | | |
| Study design | 5 | Present key elements of study design early in the paper | N | Y | Y | N | Y |
| Setting | 6 | Describe the setting, locations, and relevant dates, including periods of recruitment, exposure, follow-up, and data collection | Y | Y | Y | Y | Y |
| Participants | 7 | (*a*) Give the eligibility criteria, and the sources and methods of selection of participants | Y | Y | Y | N | Y |
| Variables | 8 | Clearly define all outcomes, exposures, predictors, potential confounders, and effect modifiers. Give diagnostic criteria, if applicable | N | Y | Y | N | Y |
| Data sources/ measurement | 9 | For each variable of interest, give sources of data and details of methods of assessment (measurement). Describe comparability of assessment methods if there is more than one group | N | Y | Y | Y | Y |
| Bias | 10 | Describe any efforts to address potential sources of bias | N | N | N | N | N |
| Study size | 11 | Explain how the study size was arrived at | Y | Y | Y | Y | Y |
| Quantitative variables | 12 | Explain how quantitative variables were handled in the analyses. If applicable, describe which groupings were chosen and why | Y | Y | Y | Y | Y |
| Statistical methods | 13 | (*a*) Describe all statistical methods, including those used to control for confounding | Y | Y | Y | Y | Y |
|  | 14 | (*b*) Describe any methods used to examine subgroups and interactions | N | N | N | N | Y |
|  | 15 | (*c*) Explain how missing data were addressed | N | N | N | N | N |
|  | 16 | (*d*) Describe any sensitivity analyses | N | N | N | N | N |
| Results | | | | | | | |
| Participants | 17 | (a) Report numbers of individuals at each stage of study—eg numbers potentially eligible, examined for eligibility, confirmed eligible, included in the study, completing follow-up, and analysed | Y | Y | Y | Y | Y |
|  | 18 | (b) Give reasons for non-participation at each stage | N | N | N | N | N |
|  | 19 | (c) Consider use of a flow diagram | N | N | N | N | N |
| Descriptive data | 20 | (a) Give characteristics of study participants (eg demographic, clinical, social) and information on exposures and potential confounders | Y | N | Y | Y | Y |
|  | 21 | (b) Indicate number of participants with missing data for each variable of interest | N | N | N | N | N |
| Outcome data | 22 | Report numbers of outcome events or summary measures | Y | Y | Y | Y | Y |
| Main results | 23 | (*a*) Give unadjusted estimates and, if applicable, confounder-adjusted estimates and their precision (eg, 95% confidence interval). Make clear which confounders were adjusted for and why they were included | N | Y | N | N | Y |
| Other analyses | 24 | Report other analyses done—eg analyses of subgroups and interactions, and sensitivity analyses | N | N | N | N | Y |
| Discussion | | | | | | | |
| Key results | 25 | Summarise key results with reference to study objectives | Y | Y | Y | Y | Y |
| Limitations | 26 | Discuss limitations of the study, taking into account sources of potential bias or imprecision. Discuss both direction and magnitude of any potential bias | N | Y | Y | N | Y |
| Interpretation | 27 | Give a cautious overall interpretation of results considering objectives, limitations, multiplicity of analyses, results from similar studies, and other relevant evidence | Y | Y | Y | Y | Y |
| Generalisability | 28 | Discuss the generalisability (external validity) of the study results | N | Y | N | N | N |
| Other information | | | | | | | |
| Funding | 29 | Give the source of funding and the role of the funders for the present study and, if applicable, for the original study on which the present article is based | N | Y | Y | N | Y |
|  |  | Summary of items 1-29 | 13 | 20 | 18 | 14 | 21 |

*Give information separately for exposed and unexposed groups.
