# Supplementary material for: The prevalence of asymptomatic neurosyphilis among HIV-negative serofast patients in China: A meta-analysis
Source: PLoS One. 2020 Nov 4;15(11):e0241572. doi: 10.1371/journal.pone.0241572 (PMC7641405; doi:10.1371/journal.pone.0241572)
Supplement: S1 Protocol — (DOCX) [file pone.0241572.s003.docx]

**中国HIV阴性梅毒血清固定患者中无症状神经梅毒流行率**

**Meta分析方案**

**Meta-analysis protocol for the prevalence of asymptomatic neurosyphilis among HIV-negative serofast patients in China**

**一、目的**

1、通过meta分析，得到中国HIV阴性的血清固定梅毒患者中无症状神经梅毒的流行情况。

2、为指导医生对血清固定的梅毒患者的临床管理提供信息。

3、了解神经梅毒与血清固定状态的关系。

**二、研究对象**

接受了脑脊液检测的HIV阴性的血清固定梅毒患者。

1. 血清固定定义：是指梅毒患者接受初次治疗之后非梅毒螺旋体血清学试验的血清滴度下降4倍及以上，随访1年及以上血清滴度仍保持低水平，未转阴。
2. 无症状神经梅毒定义：分为确诊病例和疑似病例。确诊病例的定义是无神经梅毒的临床症状，脑脊液VDRL检测结果阳性。疑似病例的定义是无神经梅毒的临床症状，但脑脊液蛋白升高（＞50mg/dL^2^）或白细胞计数（＞5wbc/mm^3^），且没有其他已知原因导致这些异常。

**三、文献检索**

1、检索文献数据库：

英文数据库：PubMed、Embase、Medline、the Cochrane library

中文数据库：中国知网、维普、万方、中国生物医学文献数据库

2、检索关键词

英文关键词：Serofast; Seroresistance; neurosyphilis; cerebrospinal fluid; China

中文关键词：神经梅毒；脑脊液；血清固定；血清抵抗

**四、文献纳入与排除标准**

1、纳入标准：

（1）研究报告了血清固定患者中神经梅毒患者的比例，或血清固定患者的脑脊液检测结果。

（2）所有血清固定患者均至少接受过一次治疗，无神经系统相关症状或体征，均接受腰椎穿刺获得了脑脊液的检测结果。

（3）研究纳入的血清固定患者的数量≥20人。

（4）研究范围限定在中国大陆。

2、排除标准：

（1）HIV阳性患者的研究被排除。

（2）对无症状神经梅毒或血清固定患者的定义或诊断标准不明确的研究被排除。

（3）综述、病例对照研究和书籍章节、会议文章被排除。

**五、文献质量评价**

采用《[STROBE声明：观察性研究报告规范](http://www.baidu.com/link?url=7YqgSel5w66lKRVfogHmN9GM4uEm2j0uykGj-guZm9fDWuZPw_2TqGjsl674ydyZqzxChiPb9MArDQ3uz2aCE4TQfNOVRY7lPp5yUbxH1tq)》对符合要求的研究进行质量评价。STROBE共29个条目，分为摘要、背景、方法学、结果、讨论、其他信息部分。

**六、数据提取**

提取信息：第一作者、发表年份、研究开展时间、脑脊液检测方法、血清固定梅毒患者数和无症状神经梅毒病例、性别、年龄、研究地区、血清固定的RPR滴度、随访时间。

**七、效应指标**

1、HIV阴性的血清固定梅毒患者中无症状神经梅毒的患病率。

2、通过meta分析，得到合并患病率。

**八、异质性检验与效应模型**

1、使用固定效应模型和随机效应模型，使用R version 3.6.2/R studio1.2.1335统计软件包META version 4.9-9计算和绘制森林图。

2、Higgins不一致性检验（I^2^）被用来评估异质性和不同研究中观察到的变异百分比。利用亚组分析进一步研究了异质性的潜在来源。

3、亚组分析的影响因素包括研究地区、病例分类和随访时间。

**九、偏倚分析**

1、使用漏斗图（funnel plot）方法来识别meta分析结果是否存在偏倚。

2、使用Egger’ test检测潜在的发表偏倚，P<0.05可以认为具有统计学意义。

**十、撰写报告**
