# Supplementary material for: The prevalence of asymptomatic neurosyphilis among HIV-negative serofast patients in China: A meta-analysis
Source: PLoS One. 2020 Nov 4;15(11):e0241572. doi: 10.1371/journal.pone.0241572 (PMC7641405; doi:10.1371/journal.pone.0241572)
Supplement: S1 Appendix — (DOCX) [file pone.0241572.s004.docx]

**Search strategy**

# English database

# Pubmed search:

(((serofast) OR (seroresistance)) AND ((neurosyphilis) OR (cerebrospinal fluid))) AND (China)

# Embase search:

('serofast' OR 'seroresistance') AND ('neurosyphilis' OR 'cerebrospinal fluid') AND ('China')

# Medline search:

(serofast or seroresistance) and (neurosyphilis or 'cerebrospinal fluid') and (China)

# Chinese database

# China National Knowledge Infrastructure (CNKI) search:

(篇关摘=神经梅毒 + 脑脊液) AND (篇关摘=血清固定 + 血清抵抗)

# Wan Fang search：

题名或关键词:((血清抵抗)+(血清固定))*题名或关键词:((神经梅毒)+(脑脊液))

# VIP search:

U=(神经梅毒 OR 脑脊液) AND U=(血清固定 OR 血清抵抗)
