# Supplementary figures and images for: The prevalence of asymptomatic neurosyphilis among HIV-negative serofast patients in China: A meta-analysis
Source: PLoS One. 2020 Nov 4;15(11):e0241572. doi: 10.1371/journal.pone.0241572 (PMC7641405; doi:10.1371/journal.pone.0241572)

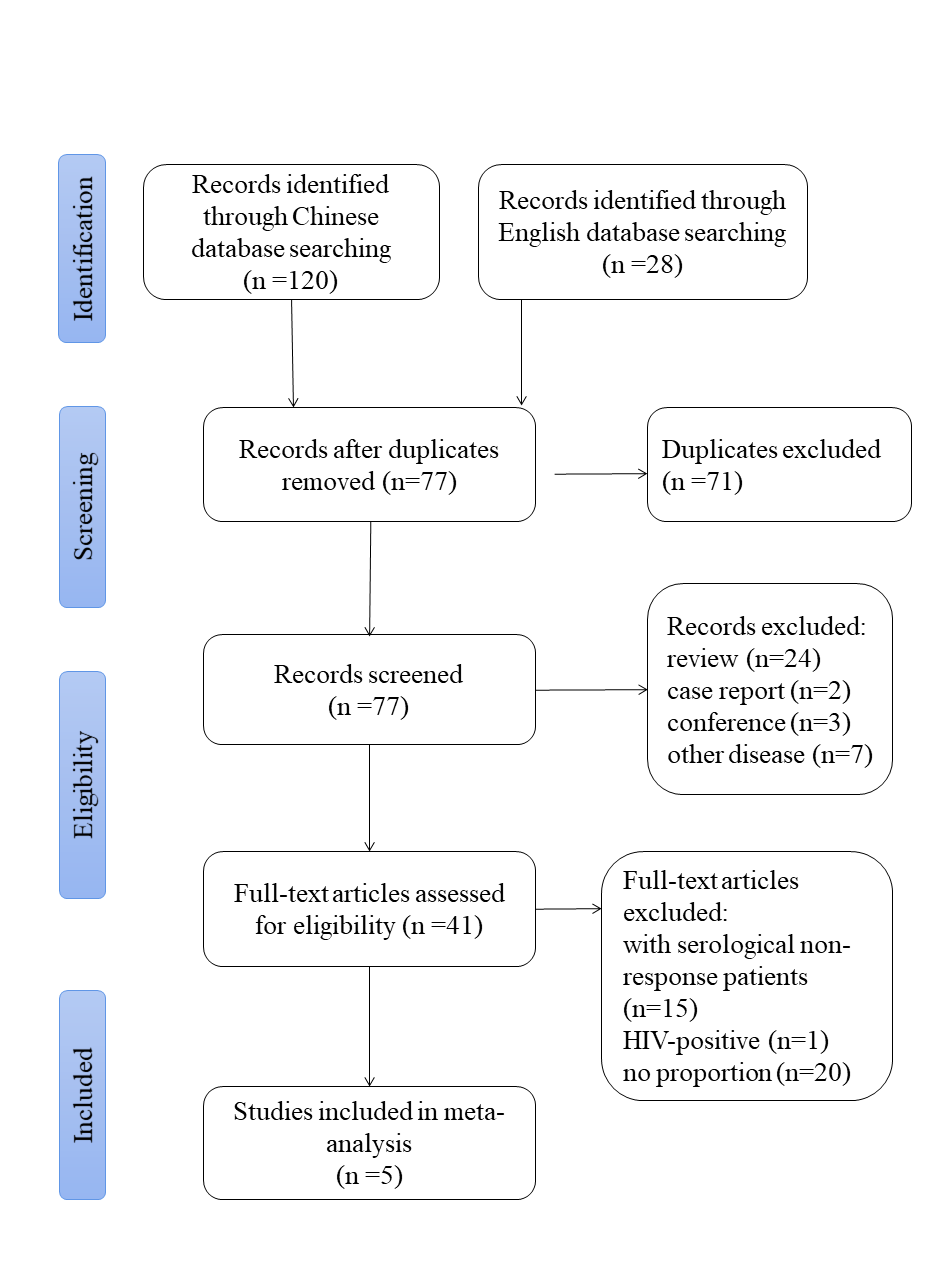

Supplement: S1 Fig — (TIF) [file pone.0241572.s005.tif]

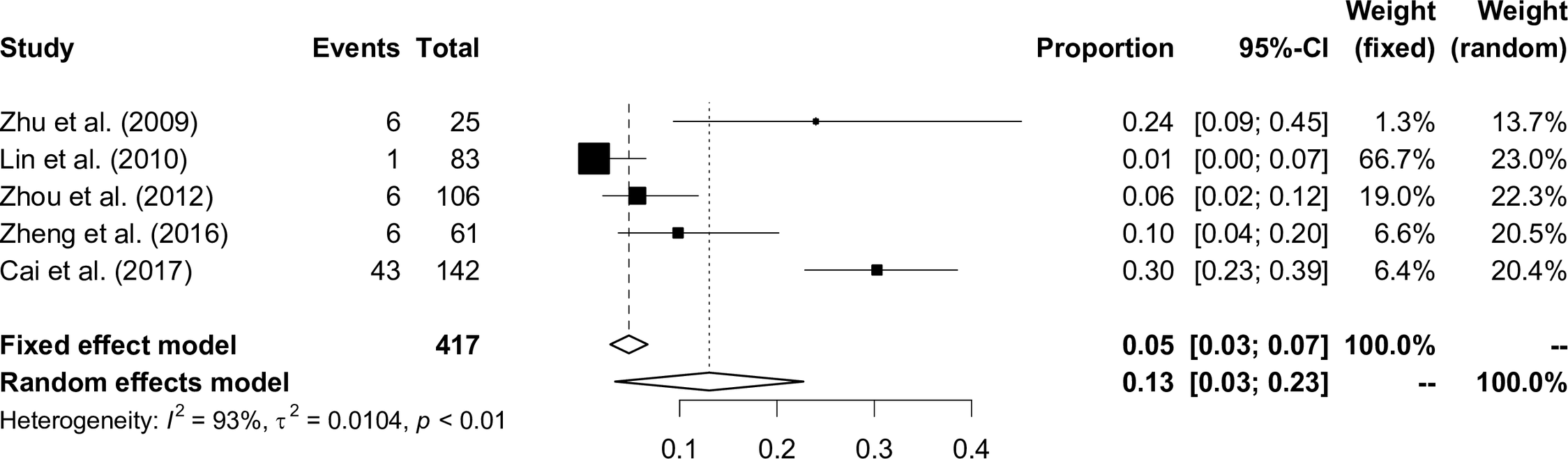

Supplement: S2 Fig — (TIF) [file pone.0241572.s006.tif]

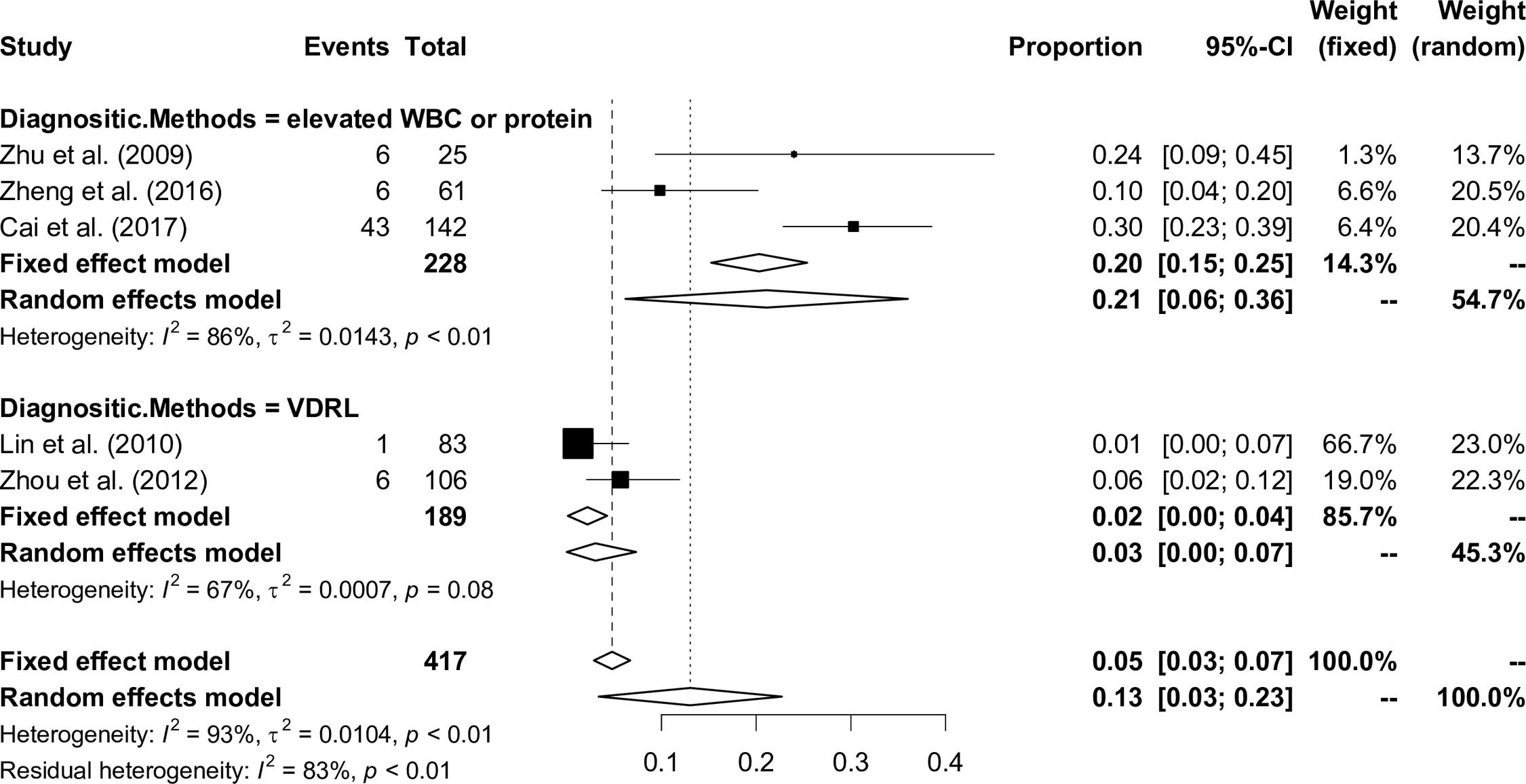

Supplement: S3 Fig — (TIF) [file pone.0241572.s007.tif]

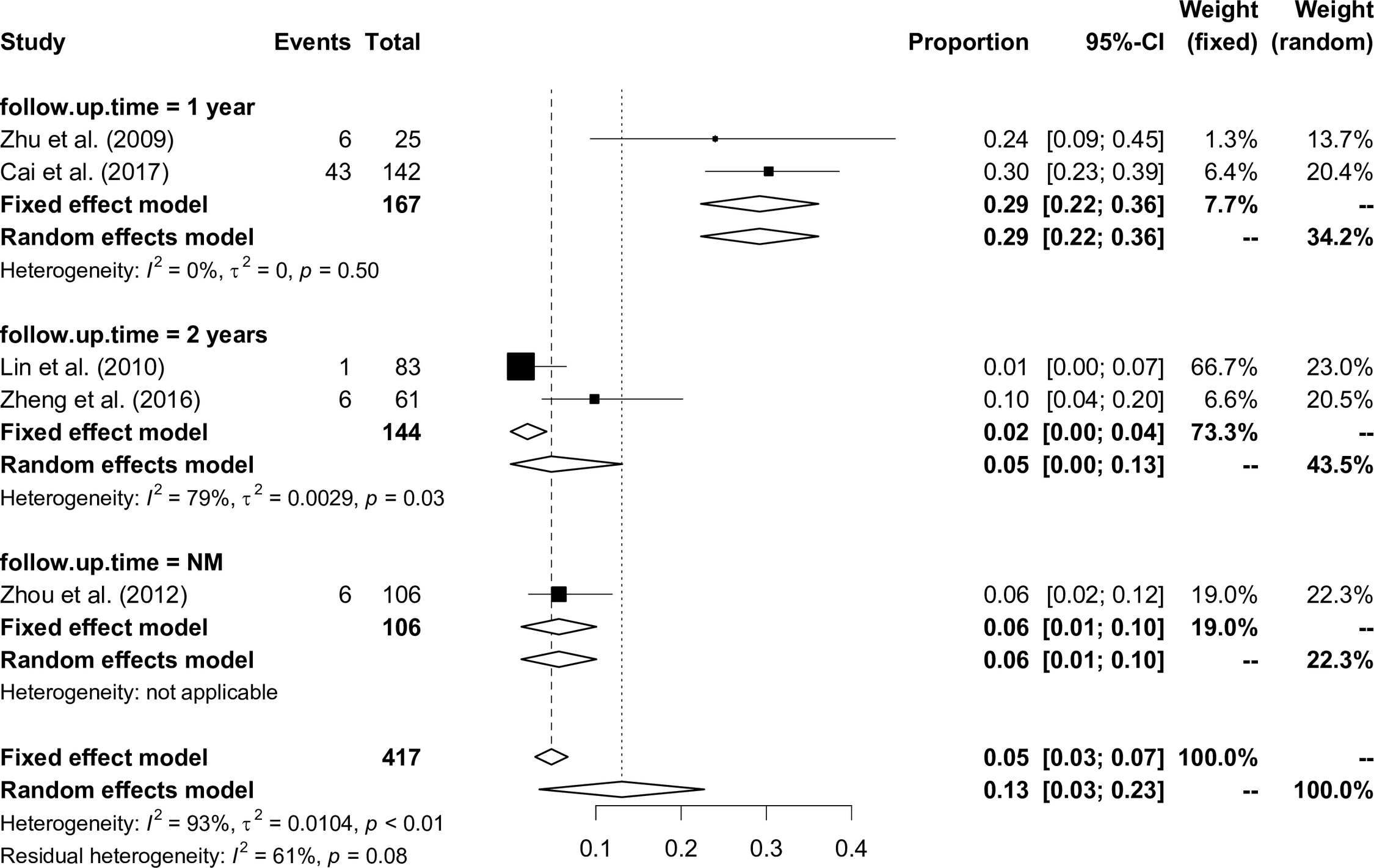

Supplement: S4 Fig — (TIF) [file pone.0241572.s008.tif]

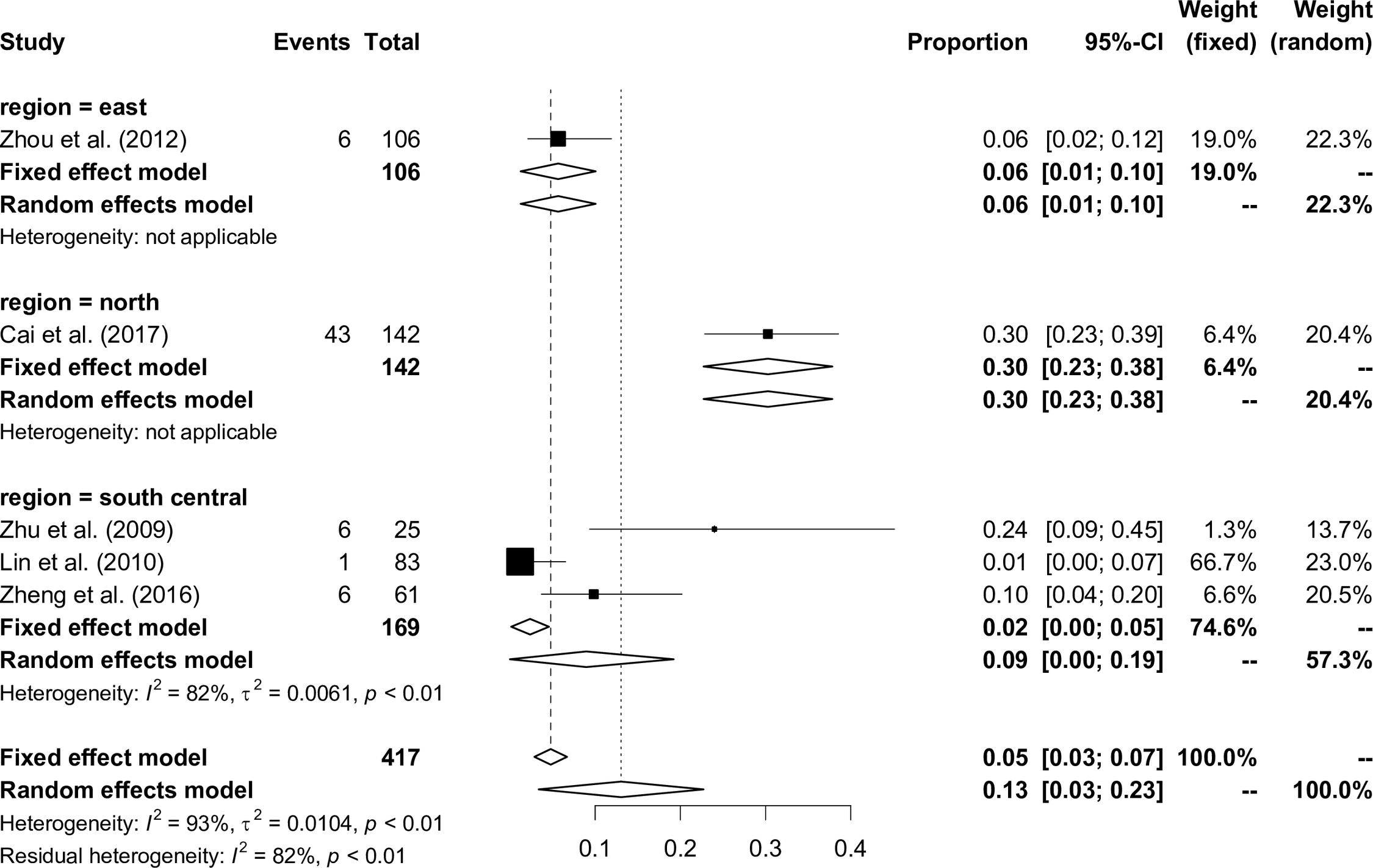

Supplement: S5 Fig — (TIF) [file pone.0241572.s009.tif]

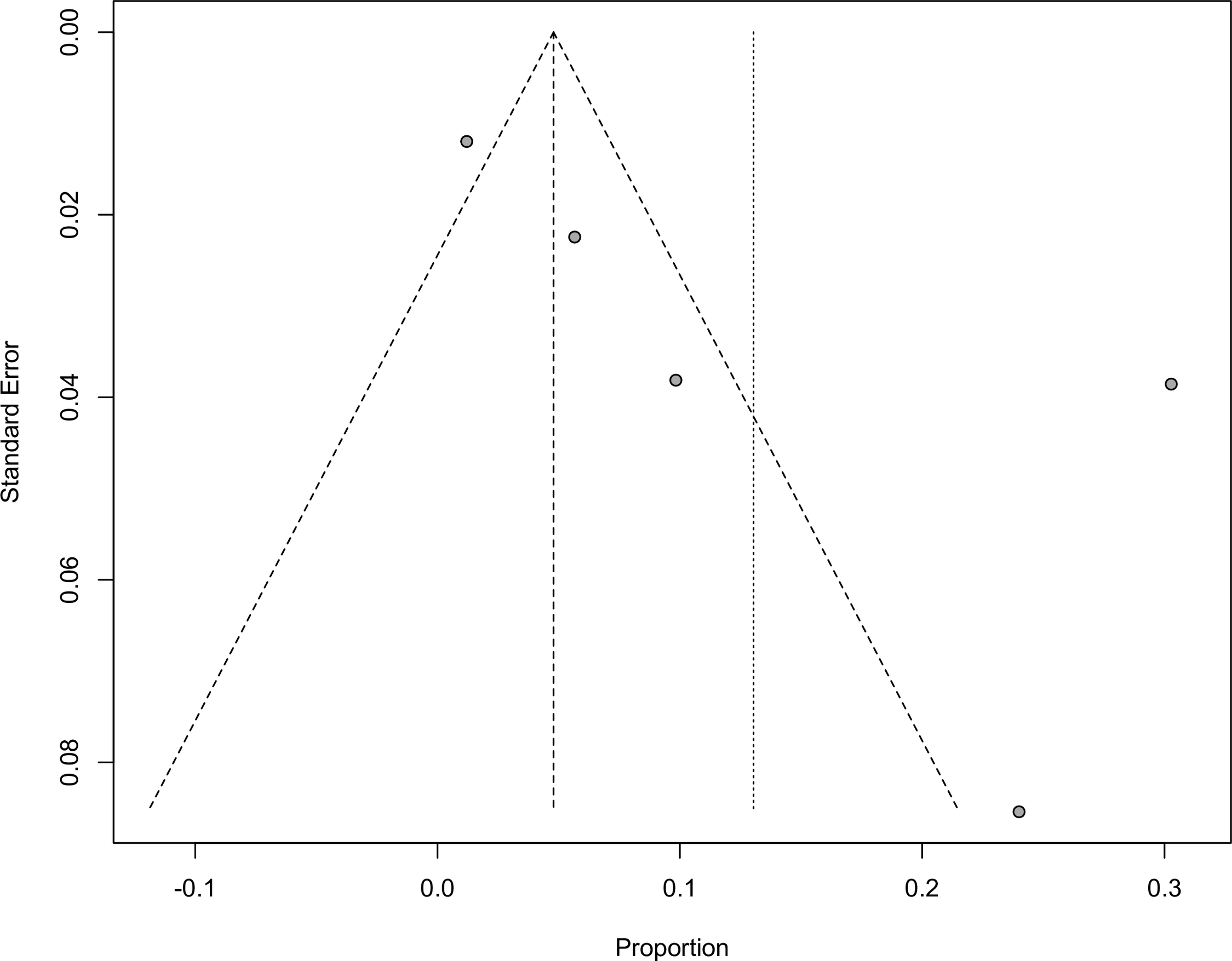

Supplement: S6 Fig — (TIF) [file pone.0241572.s010.tif]
