# Supplementary material for: The prevalence of asymptomatic neurosyphilis among HIV-negative serofast patients in China: A meta-analysis
Source: PLoS One. 2020 Nov 4;15(11):e0241572. doi: 10.1371/journal.pone.0241572 (PMC7641405; doi:10.1371/journal.pone.0241572)
Supplement: Attachment — Submitted filename: Response to Reviewers.docx [file pone.0241572.s011.docx]

# Dear Sir or Madam:

Firstly, we would like to thank you for your kind letter and for reviewers’ constructive comments concerning our article (The prevalence of asymptomatic neurosyphilis among HIV-negative serofast patients in China: a meta-analysis). These comments are all valuable and helpful for improving our article. All the authors have seriously discussed about all these comments. According to the reviewers’ comments, we have tried best to modify our manuscript to meet with the requirements of your journal.

We submitted two separate additional files about the search strategy and study protocol. Reviewers’ comments and our responses are listed below.

Major comments:

Q1. In the “Introduction” section, the author well explained the knowledge gap about current research on the prevalence of ANS among sero-fast patients with syphilis, but there was no information about the current research in China. Considering this article focusing on the prevalence in China, it would be better if the authors give more information about the current researches on this issue and its importance in China. Meanwhile, according to the article’s reference, it’s quite clear that limited recent researches focus on this issue. But it would be nice if the authors could address the worldwide and Chinese researches’ limitations on this issue clearly in the “Introduction” section.

A1: Thank you for your kind comments. We have added the related contents in the introduction section in the revised manuscript.

Q2. The way of applying AHRQ and STROBE checklists in this study seems a little bit confusing. In the “Methods” section, from a reader’s perspective, AHRQ and STROBE checklists used for quality assessment meant to assess articles based on the cross-sectional study and observational study, respectively. However, in the “Results” section, page 12 lines 165-166, these two checklists are used to examine the reporting quality and methodological qualities of all articles regardless of their types. It would be nice if the author could clarify the exact way of applying these two checklists in the methods section.

A2: Thank you for your good comments. We have removed the part of AHRQ checklist result in the revised manuscript.

In our initial submission, we used STROBE checklist and AHRQ recommended checklist for cross-sectional studies for quality assessment. In the process of revision, we think the later one was a supplement to the methodology of the study. Considering that we have adopted strictly diagnostic criteria to ensure the diagnostic accuracy, we believe it is enough for our study to adopt the STROBE checklist for cross-sectional studies only.

Minor comments：

Q1. There is no information in the “Methods” section about whether the study protocol exists, which is required by PLOS ONE.

A1: Thank you for your good comment. We have added the related contents in the “Methods” section.

Before conducting the Meta analysis, we have developed a protocol draft in Chinese and carried out data collection and analysis according to this protocol. We uploaded the Chinese version of the protocol framework as an additional file.

Q2. On page 22, “study design’s flowchart” shows one step called “studies included in qualitative synthesis”. However, this step does not be mentioned in the “Methods” section. Please give more information about it.

A2: Sorry, this is our mistake for describing the term in English. We modified this mistake in the flow chart.

Q3.  In the “Results” section, the order of sub-sections named “study characteristics” and “quality assessment” is kind of reverse. The logical line in the “Results” section is to introduce the results of paper selections at first, then studies’ characteristics and detailed analysis. From this perspective, “quality assessment” results should be put first, then the article shows the studies’ characteristics’ analysis.

A3: Thank you for your good suggestion. We adjusted the logical order of the result section in the revised manuscript.

Q4. In the “Discussion” section, page 14 lines 212 to 225, it seems that this paragraph aims to discuss the importance of the subgroup’s analysis for the future researches. It would much easier for readers to follow if the author could put a leading sentence at the beginning of this paragraph.

A4: Thank you for your good comment. We have added the leading sentence at the beginning of this paragraph in the revised manuscript.

The leading sentence is “Subgroup analysis showed follow-up time and case classification definition had great impact on the pooled prevalence. Future research should be focused on developing or employing a standardized definition of syphilis serofast and case classification criteria of ANS”.

Q5. In the “Discussion” section, lines 198, it seems the first sentence does not link to the rest part of this paragraph. If the author still wants to keep this sentence, it might be added after “Two problems always …” and be rephrased into a new one, which can explain how this study deal with the problem mentioned above as the first meta-analysis study in China.

A5: Thank you for your good suggestion. We have deleted the first sentence in the revised manuscript.

Q6. In the “Discussion” section, lines 223, the spelling of “indicat” is wrong. Please check the words spelling, punctuation, and grammar before the publication.

A6: We are really sorry for the spelling mistake. We have modified “indicat” into “indicate” in the revised manuscript.
