# Supplementary material for: The prevalence of asymptomatic neurosyphilis among HIV-negative serofast patients in China: A meta-analysis
Source: PLoS One. 2020 Nov 4;15(11):e0241572. doi: 10.1371/journal.pone.0241572 (PMC7641405; doi:10.1371/journal.pone.0241572)
Supplement: Peer review history

# Peer review: Decision Letter 0

**DOI:** 10.1371/journal.pone.0241572.r001

**Authors:** Geng-Feng Fu

14 Sep 2020

PONE-D-20-23019

The prevalence of asymptomatic neurosyphilis among HIV-negative serofast patients in China: a meta-analysis

PLOS ONE

Dear Dr. Gong,

Thank you for submitting your manuscript to PLOS ONE. After careful consideration, we feel that it has merit but does not fully meet PLOS ONE’s publication criteria as it currently stands. Therefore, we invite you to submit a revised version of the manuscript that addresses the points raised during the review process.

Please submit your revised manuscript by Oct 29 2020 11:59PM. If you will need more time than this to complete your revisions, please reply to this message or contact the journal office at plosone@plos.org. When you're ready to submit your revision, log on to https://www.editorialmanager.com/pone/ and select the 'Submissions Needing Revision' folder to locate your manuscript file.

Please include the following items when submitting your revised manuscript:

- A rebuttal letter that responds to each point raised by the academic editor and reviewer(s). You should upload this letter as a separate file labeled 'Response to Reviewers'.
- A marked-up copy of your manuscript that highlights changes made to the original version. You should upload this as a separate file labeled 'Revised Manuscript with Track Changes'.
- An unmarked version of your revised paper without tracked changes. You should upload this as a separate file labeled 'Manuscript'.

If you would like to make changes to your financial disclosure, please include your updated statement in your cover letter. Guidelines for resubmitting your figure files are available below the reviewer comments at the end of this letter.

If applicable, we recommend that you deposit your laboratory protocols in protocols.io to enhance the reproducibility of your results. Protocols.io assigns your protocol its own identifier (DOI) so that it can be cited independently in the future. For instructions see: http://journals.plos.org/plosone/s/submission-guidelines#loc-laboratory-protocols

We look forward to receiving your revised manuscript.

Kind regards,

Geng-Feng Fu, Ph.D.

Academic Editor

PLOS ONE

Journal Requirements:

When submitting your revision, we need you to address these additional requirements.

1. Please ensure that your manuscript meets PLOS ONE's style requirements, including those for file naming. The PLOS ONE style templates can be found at

https://journals.plos.org/plosone/s/file?id=wjVg/PLOSOne_formatting_sample_main_body.pdf and

https://journals.plos.org/plosone/s/file?id=ba62/PLOSOne_formatting_sample_title_authors_affiliations.pdf

2. In your Methods section, please ensure you have described your study in sufficient detail so that this revuew and analysis could be performed again. Please ensure you have stated the exact date sof last search, ensuring that this was performed recently enought to allow the inclusion of studies published in the last 12 months. In addition, please ensure you have included the full electronic search strategy for at least one database and uploaded it as an additional file.

3. PLOS requires an ORCID iD for the corresponding author in Editorial Manager on papers submitted after December 6th, 2016. Please ensure that you have an ORCID iD and that it is validated in Editorial Manager. To do this, go to ‘Update my Information’ (in the upper left-hand corner of the main menu), and click on the Fetch/Validate link next to the ORCID field. This will take you to the ORCID site and allow you to create a new iD or authenticate a pre-existing iD in Editorial Manager. Please see the following video for instructions on linking an ORCID iD to your Editorial Manager account: https://www.youtube.com/watch?v=_xcclfuvtxQ

Additional Editor Comments (if provided):

General comments:

This paper researched the prevalence of asymptomatic neurosyphilis (ANS) among HIV- negative sero-fast syphilis patients in China and its associated factors by conducting a meta-analysis. Analyzing 5 previous articles, the article concluded that the prevalence of ANS in China was high.

This article’s study design and results are sound, and its finding provides evidence to develop the Chinese clinical management for HIV-negative serofast patients and guide future researches on this issue. Additionally, here are some comments and suggestions for the author’s consideration.

Major comments:

1. In the “Introduction” section, the author well explained the knowledge gap about current research on the prevalence of ANS among sero-fast patients with syphilis, but there was no information about the current research in China. Considering this article focusing on the prevalence in China, it would be better if the authors give more information about the current researches on this issue and its importance in China. Meanwhile, according to the article’s reference, it’s quite clear that limited recent researches focus on this issue. But it would be nice if the authors could address the worldwide and Chinese researches’ limitations on this issue clearly in the “Introduction” section.

2. The way of applying AHRQ and STROBE checklists in this study seems a little bit confusing. In the “Methods” section, from a reader’s perspective, AHRQ and STROBE checklists used for quality assessment meant to assess articles based on the cross-sectional study and observational study, respectively. However, in the “Results” section, page 12 lines 165-166, these two checklists are used to examine the reporting quality and methodological qualities of all articles regardless of their types. It would be nice if the author could clarify the exact way of applying these two checklists in the methods section.

Minor comments:

1. There is no information in the” Methods” section about whether the study protocol exists, which is required by PLOS ONE.

2. On page 22, “study design’s flowchart” shows one step called “studies included in qualitative synthesis”. However, this step does not be mentioned in the “Methods” section. Please give more information about it.

3. In the “Results” section, the order of sub-sections named “study characteristics” and “quality assessment” is kind of reverse. The logical line in the “Results” section is to introduce the results of paper selections at first, then studies’ characteristics and detailed analysis. From this perspective, “quality assessment” results should be put first, then the article shows the studies’ characteristics’ analysis.

4. In the “Discussion” section, page 14 lines 212 to 225, it seems that this paragraph aims to discuss the importance of the subgroup’s analysis for the future researches. It would much easier for readers to follow if the author could put a leading sentence at the beginning of this paragraph.

5. In the “Discussion” section, lines 198, it seems the first sentence does not link to the rest part of this paragraph. If the author still wants to keep this sentence, it might be added after “Two problems always …” and be rephrased into a new one, which can explain how this study deal with the problem mentioned above as the first meta-analysis study in China.

6. In the “Discussion” section, lines 223, the spelling of “indicat” is wrong. Please check the words spelling, punctuation, and grammar before the publication.

# Author comment: Author response to Decision Letter 0

**DOI:** 10.1371/journal.pone.0241572.r002

10 Oct 2020

Dear Sir or Madam:

Firstly, we would like to thank you for your kind letter and for reviewers’ constructive comments concerning our article (The prevalence of asymptomatic neurosyphilis among HIV-negative serofast patients in China: a meta-analysis). These comments are all valuable and helpful for improving our article. All the authors have seriously discussed about all these comments. According to the reviewers’ comments, we have tried best to modify our manuscript to meet with the requirements of your journal. 

We submitted two separate additional files about the search strategy and study protocol. Reviewers’ comments and our responses are listed below. 

Major comments:

Q1. In the “Introduction” section, the author well explained the knowledge gap about current research on the prevalence of ANS among sero-fast patients with syphilis, but there was no information about the current research in China. Considering this article focusing on the prevalence in China, it would be better if the authors give more information about the current researches on this issue and its importance in China. Meanwhile, according to the article’s reference, it’s quite clear that limited recent researches focus on this issue. But it would be nice if the authors could address the worldwide and Chinese researches’ limitations on this issue clearly in the “Introduction” section.

A1: Thank you for your kind comments. We have added the related contents in the introduction section in the revised manuscript. 

Q2. The way of applying AHRQ and STROBE checklists in this study seems a little bit confusing. In the “Methods” section, from a reader’s perspective, AHRQ and STROBE checklists used for quality assessment meant to assess articles based on the cross-sectional study and observational study, respectively. However, in the “Results” section, page 12 lines 165-166, these two checklists are used to examine the reporting quality and methodological qualities of all articles regardless of their types. It would be nice if the author could clarify the exact way of applying these two checklists in the methods section.

A2: Thank you for your good comments. We have removed the part of AHRQ checklist result in the revised manuscript. 

In our initial submission, we used STROBE checklist and AHRQ recommended checklist for cross-sectional studies for quality assessment. In the process of revision, we think the later one was a supplement to the methodology of the study. Considering that we have adopted strictly diagnostic criteria to ensure the diagnostic accuracy, we believe it is enough for our study to adopt the STROBE checklist for cross-sectional studies only. 

Minor comments：

Q1. There is no information in the “Methods” section about whether the study protocol exists, which is required by PLOS ONE.

A1: Thank you for your good comment. We have added the related contents in the “Methods” section. 

Before conducting the Meta analysis, we have developed a protocol draft in Chinese and carried out data collection and analysis according to this protocol. We uploaded the Chinese version of the protocol framework as an additional file.

Q2. On page 22, “study design’s flowchart” shows one step called “studies included in qualitative synthesis”. However, this step does not be mentioned in the “Methods” section. Please give more information about it.

A2: Sorry, this is our mistake for describing the term in English. We modified this mistake in the flow chart.

Q3.  In the “Results” section, the order of sub-sections named “study characteristics” and “quality assessment” is kind of reverse. The logical line in the “Results” section is to introduce the results of paper selections at first, then studies’ characteristics and detailed analysis. From this perspective, “quality assessment” results should be put first, then the article shows the studies’ characteristics’ analysis.

A3: Thank you for your good suggestion. We adjusted the logical order of the result section in the revised manuscript.

Q4. In the “Discussion” section, page 14 lines 212 to 225, it seems that this paragraph aims to discuss the importance of the subgroup’s analysis for the future researches. It would much easier for readers to follow if the author could put a leading sentence at the beginning of this paragraph.

A4: Thank you for your good comment. We have added the leading sentence at the beginning of this paragraph in the revised manuscript.

The leading sentence is “Subgroup analysis showed follow-up time and case classification definition had great impact on the pooled prevalence. Future research should be focused on developing or employing a standardized definition of syphilis serofast and case classification criteria of ANS”.

Q5. In the “Discussion” section, lines 198, it seems the first sentence does not link to the rest part of this paragraph. If the author still wants to keep this sentence, it might be added after “Two problems always …” and be rephrased into a new one, which can explain how this study deal with the problem mentioned above as the first meta-analysis study in China.

A5: Thank you for your good suggestion. We have deleted the first sentence in the revised manuscript.

Q6. In the “Discussion” section, lines 223, the spelling of “indicat” is wrong. Please check the words spelling, punctuation, and grammar before the publication.

A6: We are really sorry for the spelling mistake. We have modified “indicat” into “indicate” in the revised manuscript.

Attachment Submitted filename: Response to Reviewers.docx Click here for additional data file.

# Editor report: Decision Letter 1

**DOI:** 10.1371/journal.pone.0241572.r003

**Authors:** Geng-Feng Fu

19 Oct 2020

The prevalence of asymptomatic neurosyphilis among HIV-negative serofast patients in China: a meta-analysis

PONE-D-20-23019R1

Dear Dr. Gong,

We’re pleased to inform you that your manuscript has been judged scientifically suitable for publication and will be formally accepted for publication once it meets all outstanding technical requirements.

Within one week, you’ll receive an e-mail detailing the required amendments. When these have been addressed, you’ll receive a formal acceptance letter and your manuscript will be scheduled for publication.

An invoice for payment will follow shortly after the formal acceptance. To ensure an efficient process, please log into Editorial Manager at http://www.editorialmanager.com/pone/, click the 'Update My Information' link at the top of the page, and double check that your user information is up-to-date. If you have any billing related questions, please contact our Author Billing department directly at authorbilling@plos.org.

If your institution or institutions have a press office, please notify them about your upcoming paper to help maximize its impact. If they’ll be preparing press materials, please inform our press team as soon as possible -- no later than 48 hours after receiving the formal acceptance. Your manuscript will remain under strict press embargo until 2 pm Eastern Time on the date of publication. For more information, please contact onepress@plos.org.

Kind regards,

Geng-Feng Fu, Ph.D.

Academic Editor

PLOS ONE

# Editor report: Acceptance letter

**DOI:** 10.1371/journal.pone.0241572.r004

**Authors:** Geng-Feng Fu

23 Oct 2020

PONE-D-20-23019R1 

The prevalence of asymptomatic neurosyphilis among HIV-negative serofast patients in China: a meta-analysis 

Dear Dr. Gong:

I'm pleased to inform you that your manuscript has been deemed suitable for publication in PLOS ONE. Congratulations! Your manuscript is now with our production department. 

If your institution or institutions have a press office, please let them know about your upcoming paper now to help maximize its impact. If they'll be preparing press materials, please inform our press team within the next 48 hours. Your manuscript will remain under strict press embargo until 2 pm Eastern Time on the date of publication. For more information please contact onepress@plos.org.

If we can help with anything else, please email us at plosone@plos.org. 

Thank you for submitting your work to PLOS ONE and supporting open access. 

Kind regards, 

PLOS ONE Editorial Office Staff

on behalf of

Dr. Geng-Feng Fu 

Academic Editor

PLOS ONE
